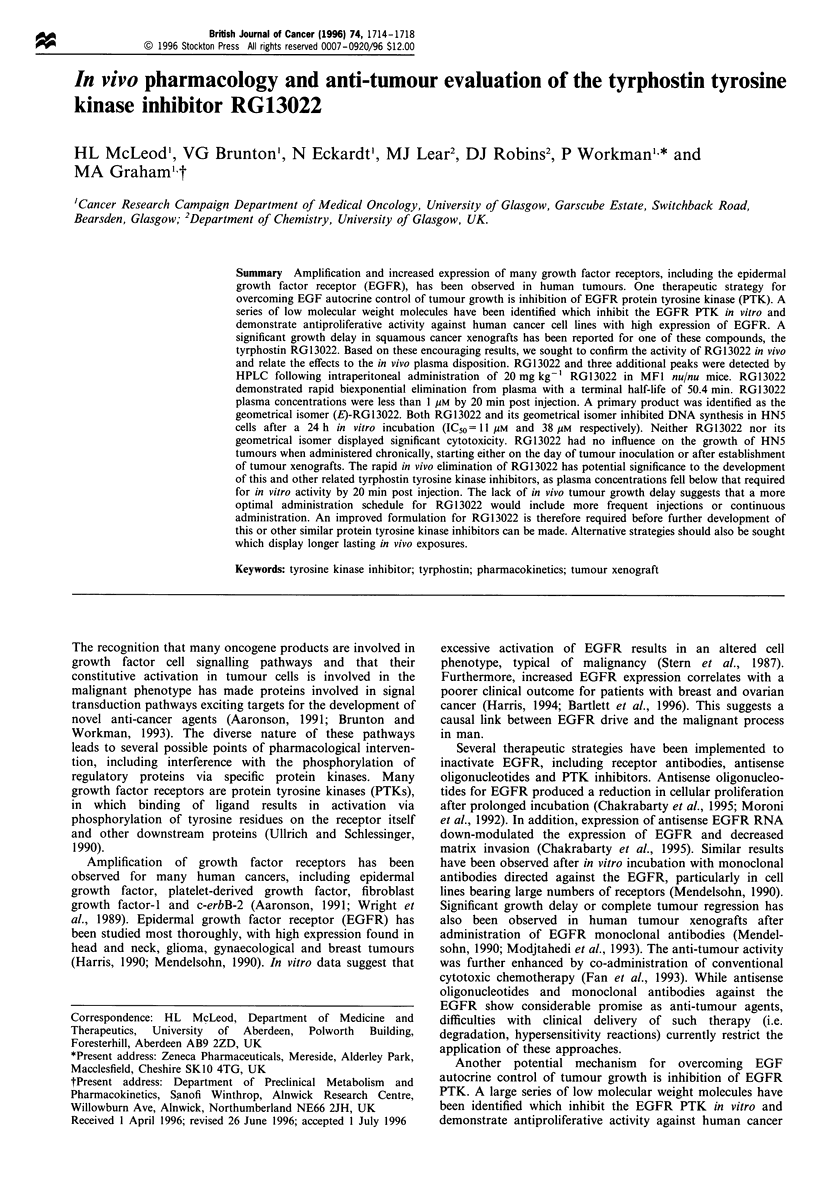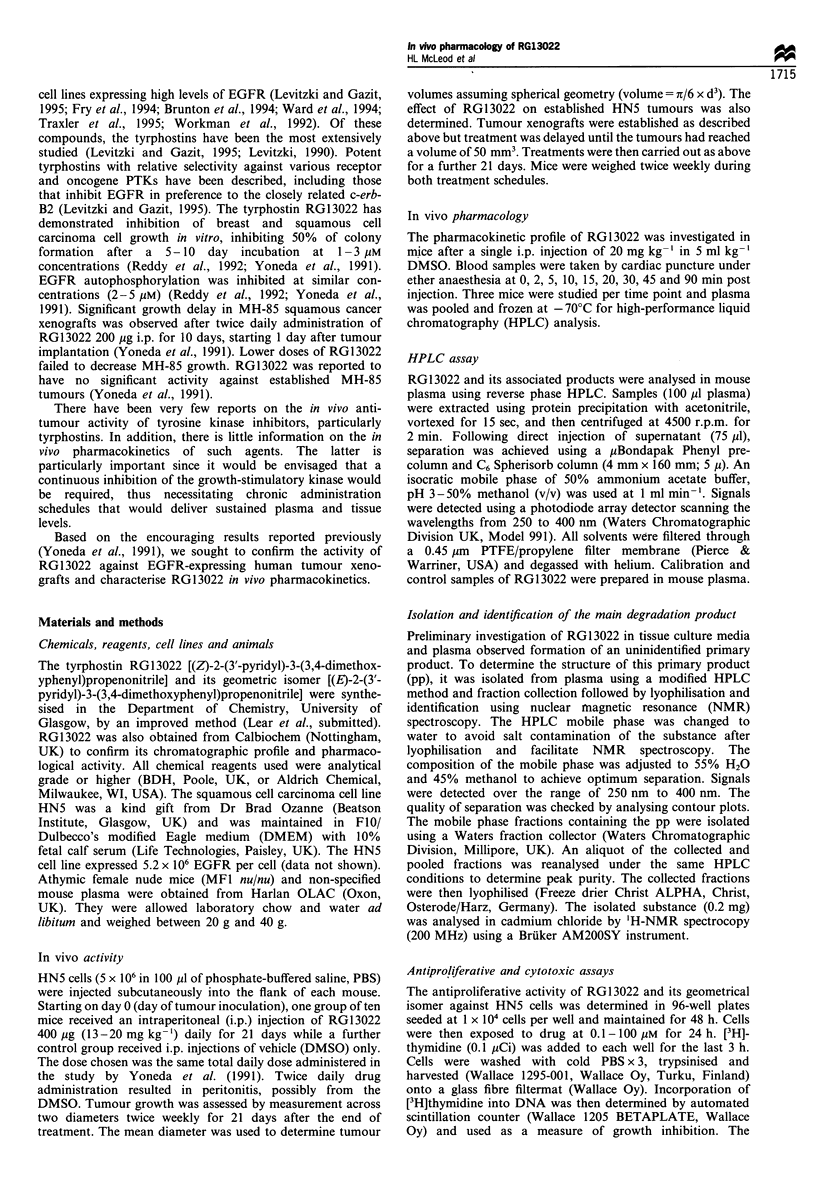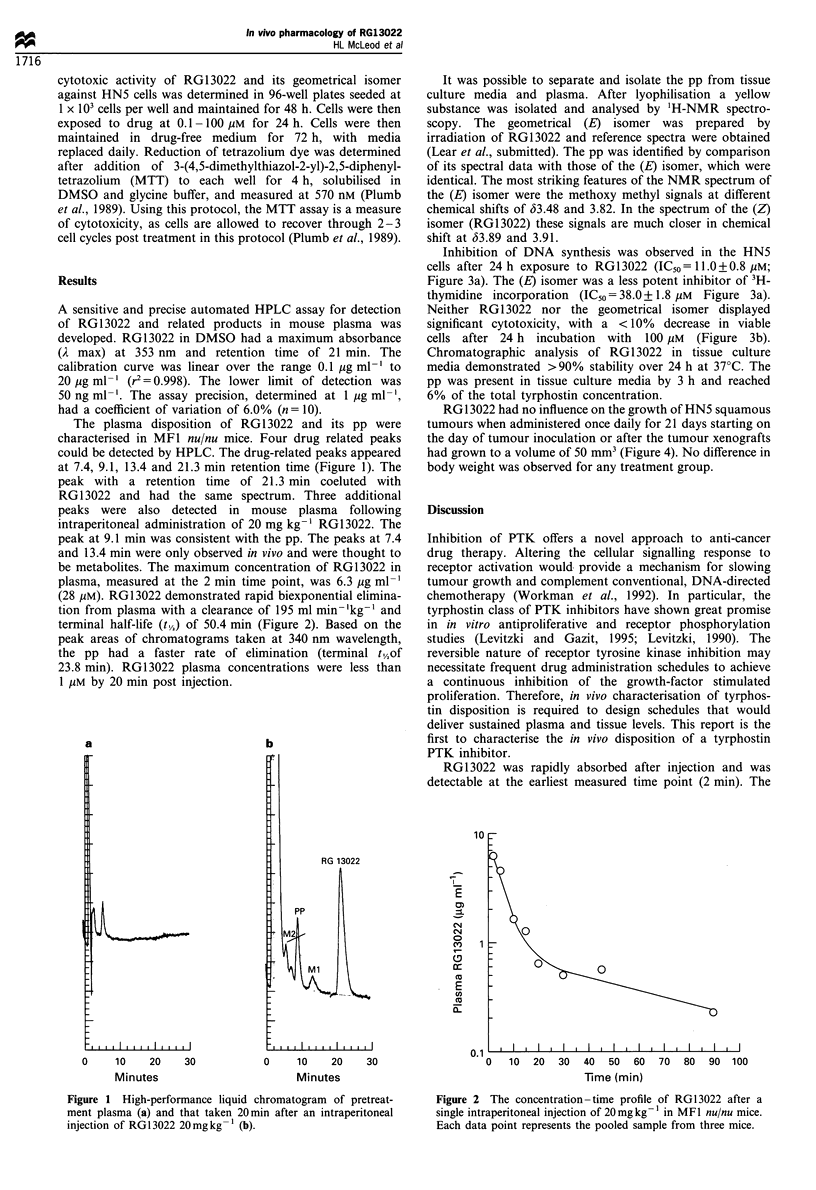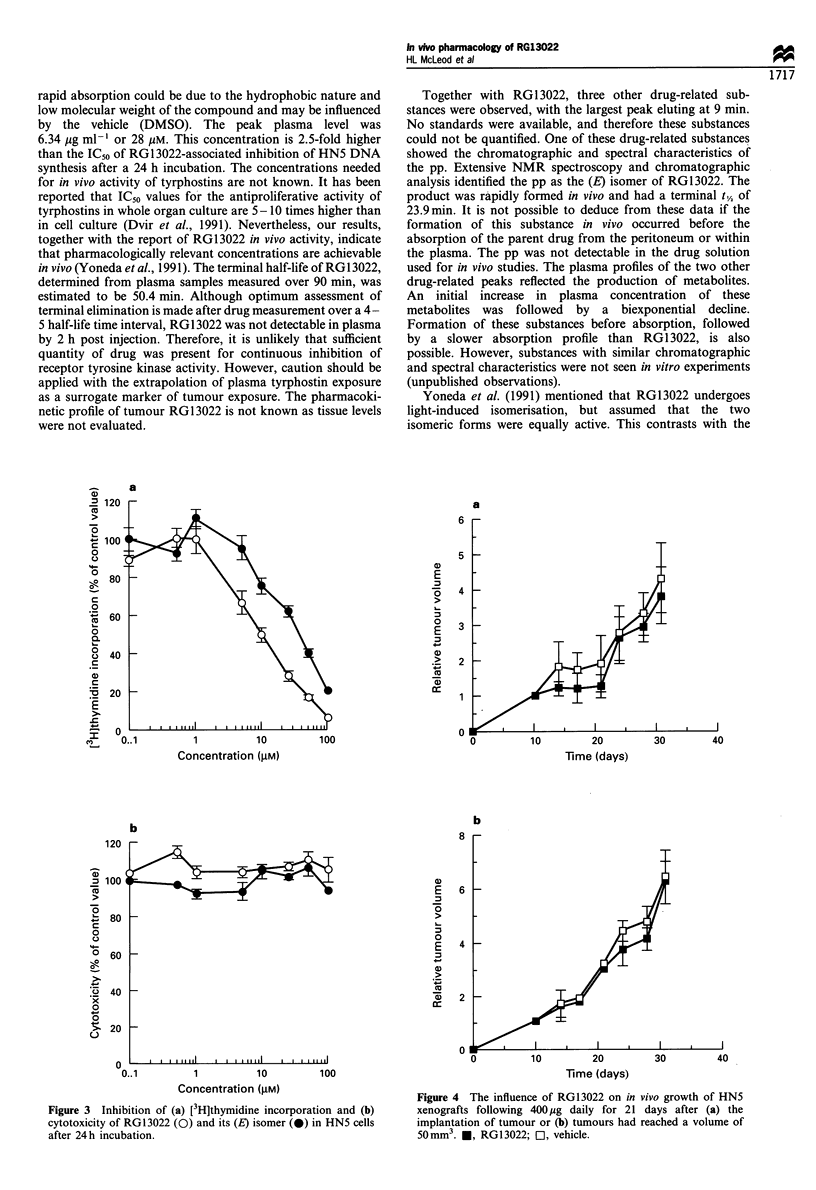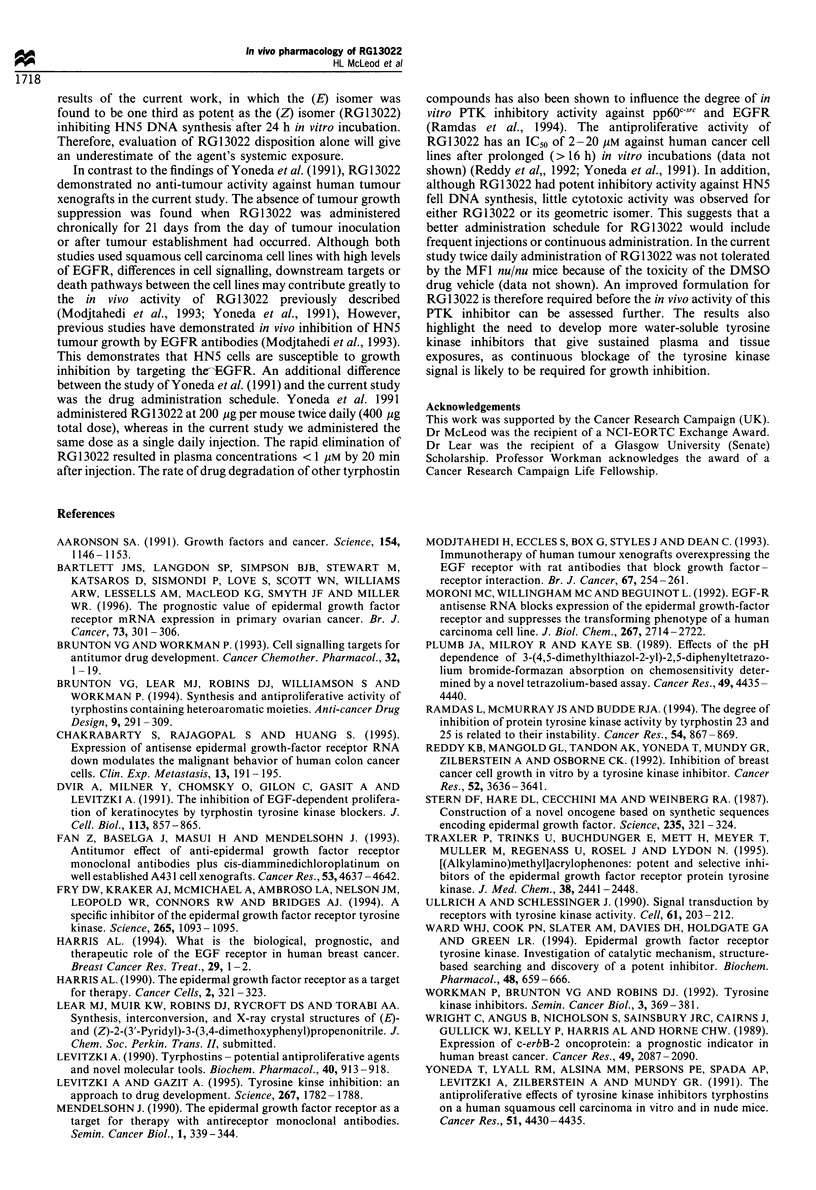# In vivo pharmacology and anti-tumour evaluation of the tyrphostin tyrosine kinase inhibitor RG13022.

**DOI:** 10.1038/bjc.1996.620

**Published:** 1996-12

**Authors:** H. L. McLeod, V. G. Brunton, N. Eckardt, M. J. Lear, D. J. Robins, P. Workman, M. A. Graham

**Affiliations:** Cancer Research Campaign Department of Medical Oncology, University of Glasgow, Bearsden, UK.

## Abstract

Amplification and increased expression of many growth factor receptors, including the epidermal growth factor receptor (EGFR), has been observed in human tumours. One therapeutic strategy for overcoming EGF autocrine control of tumour growth is inhibition of EGFR protein tyrosine kinase (PTK). A series of low molecular weight molecules have been identified which inhibit the EGFR PTK in vitro and demonstrate antiproliferative activity against human cancer cell lines with high expression of EGFR. A significant growth delay in squamous cancer xenografts has been reported for one of these compounds, the tyrphostin RG13022. Based on these encouraging results, we sought to confirm the activity of RG13022 in vivo and relate the effects to the in vivo plasma disposition. RG13022 and three additional peaks were detected by HPLC following intraperitoneal administration of 20 mg kg-1 RG13022 in MF1 nu/nu mice. RG13022 demonstrated rapid biexponential elimination from plasma with a terminal half-life of 50.4 min. RG13022 plasma concentrations were less than 1 microM by 20 min post injection. A primary product was identified as the geometrical isomer (E)-RG13022. Both RG13022 and its geometrical isomer inhibited DNA synthesis in HN5 cells after a 24 h in vitro incubation (IC50 = 11 microM and 38 microM respectively). Neither RG13022 nor its geometrical isomer displayed significant cytotoxicity. RG13022 had no influence on the growth of HN5 tumours when administered chronically, starting either on the day of tumour inoculation or after establishment of tumour xenografts. The rapid in vivo elimination of RG13022 has potential significance to the development of this and other related tyrphostin tyrosine kinase inhibitors, as plasma concentrations fell below that required for in vitro activity by 20 min post injection. The lack of in vivo tumour growth delay suggests that a more optimal administration schedule for RG13022 would include more frequent injections or continuous administration. An improved formulation for RG13022 is therefore required before further development of this or other similar protein tyrosine kinase inhibitors can be made. Alternative strategies should also be sought which display longer lasting in vivo exposures.


British Journal of Cancer (1996) 74, 1714–1718
© 1996 Stockton Press All rights reserved 0007–0920/96 $12.00

### *In vivo* pharmacology and anti-tumour evaluation of the tyrphostin tyrosine kinase inhibitor RG13022

HL McLeod[1], VG Brunton[1], N Eckardt[1], MJ Lear[2], DJ Robins[2], P Workman[1,*] and MA Graham[1,†]

[1]*Cancer Research Campaign Department of Medical Oncology, University of Glasgow, Garscube Estate, Switchback Road, Bearsden, Glasgow;* [2]*Department of Chemistry, University of Glasgow, UK.*

**Summary** Amplification and increased expression of many growth factor receptors, including the epidermal growth factor receptor (EGFR), has been observed in human tumours. One therapeutic strategy for overcoming EGF autocrine control of tumour growth is inhibition of EGFR protein tyrosine kinase (PTK). A series of low molecular weight molecules have been identified which inhibit the EGFR PTK *in vitro* and demonstrate antiproliferative activity against human cancer cell lines with high expression of EGFR. A significant growth delay in squamous cancer xenografts has been reported for one of these compounds, the tyrphostin RG13022. Based on these encouraging results, we sought to confirm the activity of RG13022 *in vivo* and relate the effects to the *in vivo* plasma disposition. RG13022 and three additional peaks were detected by HPLC following intraperitoneal administration of 20 mg $kg^{-1}$ RG13022 in MF1 *nu/nu* mice. RG13022 demonstrated rapid biexponential elimination from plasma with a terminal half-life of 50.4 min. RG13022 plasma concentrations were less than 1 $\mu$M by 20 min post injection. A primary product was identified as the geometrical isomer (*E*)-RG13022. Both RG13022 and its geometrical isomer inhibited DNA synthesis in HN5 cells after a 24 h *in vitro* incubation ($IC_{50}$ = 11 $\mu$M and 38 $\mu$M respectively). Neither RG13022 nor its geometrical isomer displayed significant cytotoxicity. RG13022 had no influence on the growth of HN5 tumours when administered chronically, starting either on the day of tumour inoculation or after establishment of tumour xenografts. The rapid *in vivo* elimination of RG13022 has potential significance to the development of this and other related tyrphostin tyrosine kinase inhibitors, as plasma concentrations fell below that required for *in vitro* activity by 20 min post injection. The lack of *in vivo* tumour growth delay suggests that a more optimal administration schedule for RG13022 would include more frequent injections or continuous administration. An improved formulation for RG13022 is therefore required before further development of this or other similar protein tyrosine kinase inhibitors can be made. Alternative strategies should also be sought which display longer lasting *in vivo* exposures.

**Keywords:** tyrosine kinase inhibitor; tyrphostin; pharmacokinetics; tumour xenograft

The recognition that many oncogene products are involved in growth factor cell signalling pathways and that their constitutive activation in tumour cells is involved in the malignant phenotype has made proteins involved in signal transduction pathways exciting targets for the development of novel anti-cancer agents (Aaronson, 1991; Brunton and Workman, 1993). The diverse nature of these pathways leads to several possible points of pharmacological intervention, including interference with the phosphorylation of regulatory proteins via specific protein kinases. Many growth factor receptors are protein tyrosine kinases (PTKs), in which binding of ligand results in activation via phosphorylation of tyrosine residues on the receptor itself and other downstream proteins (Ullrich and Schlessinger, 1990).

Amplification of growth factor receptors has been observed for many human cancers, including epidermal growth factor, platelet-derived growth factor, fibroblast growth factor-1 and c-*erb*B-2 (Aaronson, 1991; Wright *et al.*, 1989). Epidermal growth factor receptor (EGFR) has been studied most thoroughly, with high expression found in head and neck, glioma, gynaecological and breast tumours (Harris, 1990; Mendelsohn, 1990). *In vitro* data suggest that excessive activation of EGFR results in an altered cell phenotype, typical of malignancy (Stern *et al.*, 1987). Furthermore, increased EGFR expression correlates with a poorer clinical outcome for patients with breast and ovarian cancer (Harris, 1994; Bartlett *et al.*, 1996). This suggests a causal link between EGFR drive and the malignant process in man.

Several therapeutic strategies have been implemented to inactivate EGFR, including receptor antibodies, antisense oligonucleotides and PTK inhibitors. Antisense oligonucleotides for EGFR produced a reduction in cellular proliferation after prolonged incubation (Chakrabarty *et al.*, 1995; Moroni *et al.*, 1992). In addition, expression of antisense EGFR RNA down-modulated the expression of EGFR and decreased matrix invasion (Chakrabarty *et al.*, 1995). Similar results have been observed after *in vitro* incubation with monoclonal antibodies directed against the EGFR, particularly in cell lines bearing large numbers of receptors (Mendelsohn, 1990). Significant growth delay or complete tumour regression has also been observed in human tumour xenografts after administration of EGFR monoclonal antibodies (Mendelsohn, 1990; Modjtahedi *et al.*, 1993). The anti-tumour activity was further enhanced by co-administration of conventional cytotoxic chemotherapy (Fan *et al.*, 1993). While antisense oligonucleotides and monoclonal antibodies against the EGFR show considerable promise as anti-tumour agents, difficulties with clinical delivery of such therapy (i.e. degradation, hypersensitivity reactions) currently restrict the application of these approaches.

Another potential mechanism for overcoming EGF autocrine control of tumour growth is inhibition of EGFR PTK. A large series of low molecular weight molecules have been identified which inhibit the EGFR PTK *in vitro* and demonstrate antiproliferative activity against human cancer

Correspondence: HL McLeod, Department of Medicine and Therapeutics, University of Aberdeen, Polworth Building, Foresterhill, Aberdeen AB9 2ZD, UK

*Present address: Zeneca Pharmaceuticals, Mereside, Alderley Park, Macclesfield, Cheshire SK10 4TG, UK

†Present address: Department of Preclinical Metabolism and Pharmacokinetics, Sanofi Winthrop, Alnwick Research Centre, Willowburn Ave, Alnwick, Northumberland NE66 2JH, UK

Received 1 April 1996; revised 26 June 1996; accepted 1 July 1996

cell lines expressing high levels of EGFR (Levitzki and Gazit, 1995; Fry *et al.*, 1994; Brunton *et al.*, 1994; Ward *et al.*, 1994; Traxler *et al.*, 1995; Workman *et al.*, 1992). Of these compounds, the tyrphostins have been the most extensively studied (Levitzki and Gazit, 1995; Levitzki, 1990). Potent tyrphostins with relative selectivity against various receptor and oncogene PTKs have been described, including those that inhibit EGFR in preference to the closely related c-*erb*-B2 (Levitzki and Gazit, 1995). The tyrphostin RG13022 has demonstrated inhibition of breast and squamous cell carcinoma cell growth *in vitro*, inhibiting 50% of colony formation after a 5–10 day incubation at 1–3 $\mu$M concentrations (Reddy *et al.*, 1992; Yoneda *et al.*, 1991). EGFR autophosphorylation was inhibited at similar concentrations (2–5 $\mu$M) (Reddy *et al.*, 1992; Yoneda *et al.*, 1991). Significant growth delay in MH-85 squamous cancer xenografts was observed after twice daily administration of RG13022 200 $\mu$g i.p. for 10 days, starting 1 day after tumour implantation (Yoneda *et al.*, 1991). Lower doses of RG13022 failed to decrease MH-85 growth. RG13022 was reported to have no significant activity against established MH-85 tumours (Yoneda *et al.*, 1991).

There have been very few reports on the *in vivo* antitumour activity of tyrosine kinase inhibitors, particularly tyrphostins. In addition, there is little information on the *in vivo* pharmacokinetics of such agents. The latter is particularly important since it would be envisaged that a continuous inhibition of the growth-stimulatory kinase would be required, thus necessitating chronic administration schedules that would deliver sustained plasma and tissue levels.

Based on the encouraging results reported previously (Yoneda et al., 1991), we sought to confirm the activity of RG13022 against EGFR-expressing human tumour xenografts and characterise RG13022 *in vivo* pharmacokinetics.

### Materials and methods

### *Chemicals, reagents, cell lines and animals*

The tyrphostin RG13022 [(*Z*)-2-(3′-pyridyl)-3-(3,4-dimethoxyphenyl)propenonitrile] and its geometric isomer [(*E*)-2-(3′-pyridyl)-3-(3,4-dimethoxyphenyl)propenonitrile] were synthesised in the Department of Chemistry, University of Glasgow, by an improved method (Lear *et al.*, submitted). RG13022 was also obtained from Calbiochem (Nottingham, UK) to confirm its chromatographic profile and pharmacological activity. All chemical reagents used were analytical grade or higher (BDH, Poole, UK, or Aldrich Chemical, Milwaukee, WI, USA). The squamous cell carcinoma cell line HN5 was a kind gift from Dr Brad Ozanne (Beatson Institute, Glasgow, UK) and was maintained in F10/Dulbecco's modified Eagle medium (DMEM) with 10% fetal calf serum (Life Technologies, Paisley, UK). The HN5 cell line expressed $5.2 \times 10^6$ EGFR per cell (data not shown). Athymic female nude mice (MF1 *nu/nu*) and non-specified mouse plasma were obtained from Harlan OLAC (Oxon, UK). They were allowed laboratory chow and water *ad libitum* and weighed between 20 g and 40 g.

### In vivo *activity*

HN5 cells ($5 \times 10^6$ in 100 $\mu$l of phosphate-buffered saline, PBS) were injected subcutaneously into the flank of each mouse. Starting on day 0 (day of tumour inoculation), one group of ten mice received an intraperitoneal (i.p.) injection of RG13022 400 $\mu$g (13–20 mg $kg^{-1}$) daily for 21 days while a further control group received i.p. injections of vehicle (DMSO) only. The dose chosen was the same total daily dose administered in the study by Yoneda *et al.* (1991). Twice daily drug administration resulted in peritonitis, possibly from the DMSO. Tumour growth was assessed by measurement across two diameters twice weekly for 21 days after the end of treatment. The mean diameter was used to determine tumour volumes assuming spherical geometry (volume = $\pi/6 \times d^3$). The effect of RG13022 on established HN5 tumours was also determined. Tumour xenografts were established as described above but treatment was delayed until the tumours had reached a volume of 50 $mm^3$. Treatments were then carried out as above for a further 21 days. Mice were weighed twice weekly during both treatment schedules.

### In vivo *pharmacology*

The pharmacokinetic profile of RG13022 was investigated in mice after a single i.p. injection of 20 mg $kg^{-1}$ in 5 ml $kg^{-1}$ DMSO. Blood samples were taken by cardiac puncture under ether anaesthesia at 0, 2, 5, 10, 15, 20, 30, 45 and 90 min post injection. Three mice were studied per time point and plasma was pooled and frozen at −70°C for high-performance liquid chromatography (HPLC) analysis.

### *HPLC assay*

RG13022 and its associated products were analysed in mouse plasma using reverse phase HPLC. Samples (100 $\mu$l plasma) were extracted using protein precipitation with acetonitrile, vortexed for 15 sec, and then centrifuged at 4500 r.p.m. for 2 min. Following direct injection of supernatant (75 $\mu$l), separation was achieved using a $\mu$Bondapak Phenyl precolumn and $C_6$ Spherisorb column (4 mm × 160 mm; 5 $\mu$). An isocratic mobile phase of 50% ammonium acetate buffer, pH 3–50% methanol (v/v) was used at 1 ml $min^{-1}$. Signals were detected using a photodiode array detector scanning the wavelengths from 250 to 400 nm (Waters Chromatographic Division UK, Model 991). All solvents were filtered through a 0.45 $\mu$m PTFE/propylene filter membrane (Pierce & Warriner, USA) and degassed with helium. Calibration and control samples of RG13022 were prepared in mouse plasma.

### *Isolation and identification of the main degradation product*

Preliminary investigation of RG13022 in tissue culture media and plasma observed formation of an uninidentified primary product. To determine the structure of this primary product (pp), it was isolated from plasma using a modified HPLC method and fraction collection followed by lyophilisation and identification using nuclear magnetic resonance (NMR) spectroscopy. The HPLC mobile phase was changed to water to avoid salt contamination of the substance after lyophilisation and facilitate NMR spectroscopy. The composition of the mobile phase was adjusted to 55% $H_2O$ and 45% methanol to achieve optimum separation. Signals were detected over the range of 250 nm to 400 nm. The quality of separation was checked by analysing contour plots. The mobile phase fractions containing the pp were isolated using a Waters fraction collector (Waters Chromatographic Division, Millipore, UK). An aliquot of the collected and pooled fractions was reanalysed under the same HPLC conditions to determine peak purity. The collected fractions were then lyophilised (Freeze drier Christ ALPHA, Christ, Osterode/Harz, Germany). The isolated substance (0.2 mg) was analysed in cadmium chloride by $^1$H-NMR spectrocopy (200 MHz) using a Brüker AM200SY instrument.

### *Antiproliferative and cytotoxic assays*

The antiproliferative activity of RG13022 and its geometrical isomer against HN5 cells was determined in 96-well plates seeded at $1 \times 10^4$ cells per well and maintained for 48 h. Cells were then exposed to drug at 0.1–100 $\mu$M for 24 h. [$^3$H]-thymidine (0.1 $\mu$Ci) was added to each well for the last 3 h. Cells were washed with cold PBS × 3, trypsinised and harvested (Wallace 1295-001, Wallace Oy, Turku, Finland) onto a glass fibre filtermat (Wallace Oy). Incorporation of [$^3$H]thymidine into DNA was then determined by automated scintillation counter (Wallace 1205 BETAPLATE, Wallace Oy) and used as a measure of growth inhibition. The cytotoxic activity of RG13022 and its geometrical isomer against HN5 cells was determined in 96-well plates seeded at $1 \times 10^3$ cells per well and maintained for 48 h. Cells were then exposed to drug at 0.1 – 100 $\mu$M for 24 h. Cells were then maintained in drug-free medium for 72 h, with media replaced daily. Reduction of tetrazolium dye was determined after addition of 3-(4,5-dimethylthiazol-2-yl)-2,5-diphenyl-tetrazolium (MTT) to each well for 4 h, solubilised in DMSO and glycine buffer, and measured at 570 nM (Plumb *et al.*, 1989). Using this protocol, the MTT assay is a measure of cytotoxicity, as cells are allowed to recover through 2 – 3 cell cycles post treatment in this protocol (Plumb *et al.*, 1989).

### Results

A sensitive and precise automated HPLC assay for detection of RG13022 and related products in mouse plasma was developed. RG13022 in DMSO had a maximum absorbance ($\lambda$ max) at 353 nm and retention time of 21 min. The calibration curve was linear over the range 0.1 $\mu$g ml$^{-1}$ to 20 $\mu$g ml$^{-1}$ ($r^2 = 0.998$). The lower limit of detection was 50 ng ml$^{-1}$. The assay precision, determined at 1 $\mu$g ml$^{-1}$, had a coefficient of variation of 6.0% ($n = 10$).

The plasma disposition of RG13022 and its pp were characterised in MF1 *nu*/*nu* mice. Four drug related peaks could be detected by HPLC. The drug-related peaks appeared at 7.4, 9.1, 13.4 and 21.3 min retention time (Figure 1). The peak with a retention time of 21.3 min coeluted with RG13022 and had the same spectrum. Three additional peaks were also detected in mouse plasma following intraperitoneal administration of 20 mg kg$^{-1}$ RG13022. The peak at 9.1 min was consistent with the pp. The peaks at 7.4 and 13.4 min were only observed *in vivo* and were thought to be metabolites. The maximum concentration of RG13022 in plasma, measured at the 2 min time point, was 6.3 $\mu$g ml$^{-1}$ (28 $\mu$M). RG13022 demonstrated rapid biexponential elimination from plasma with a clearance of 195 ml min$^{-1}$kg$^{-1}$ and terminal half-life ($t_{½}$) of 50.4 min (Figure 2). Based on the peak areas of chromatograms taken at 340 nm wavelength, the pp had a faster rate of elimination (terminal $t_{½}$of 23.8 min). RG13022 plasma concentrations were less than 1 $\mu$M by 20 min post injection.

**Figure 1** High-performance liquid chromatogram of pretreatment plasma (**a**) and that taken 20 min after an intraperitoneal injection of RG13022 20 mg kg$^{-1}$ (**b**).

It was possible to separate and isolate the pp from tissue culture media and plasma. After lyophilisation a yellow substance was isolated and analysed by $^1$H-NMR spectroscopy. The geometrical (*E*) isomer was prepared by irradiation of RG13022 and reference spectra were obtained (Lear *et al.*, submitted). The pp was identified by comparison of its spectral data with those of the (*E*) isomer, which were identical. The most striking features of the NMR spectrum of the (*E*) isomer were the methoxy methyl signals at different chemical shifts of $\delta$3.48 and 3.82. In the spectrum of the (*Z*) isomer (RG13022) these signals are much closer in chemical shift at $\delta$3.89 and 3.91.

Inhibition of DNA synthesis was observed in the HN5 cells after 24 h exposure to RG13022 ($IC_{50} = 11.0 \pm 0.8$ $\mu$M; Figure 3a). The (*E*) isomer was a less potent inhibitor of $^3$H-thymidine incorporation ($IC_{50} = 38.0 \pm 1.8$ $\mu$M Figure 3a). Neither RG13022 nor the geometrical isomer displayed significant cytotoxicity, with a <10% decrease in viable cells after 24 h incubation with 100 $\mu$M (Figure 3b). Chromatographic analysis of RG13022 in tissue culture media demonstrated >90% stability over 24 h at 37°C. The pp was present in tissue culture media by 3 h and reached 6% of the total tyrphostin concentration.

RG13022 had no influence on the growth of HN5 squamous tumours when administered once daily for 21 days starting on the day of tumour inoculation or after the tumour xenografts had grown to a volume of 50 mm$^3$ (Figure 4). No difference in body weight was observed for any treatment group.

### Discussion

Inhibition of PTK offers a novel approach to anti-cancer drug therapy. Altering the cellular signalling response to receptor activation would provide a mechanism for slowing tumour growth and complement conventional, DNA-directed chemotherapy (Workman *et al.*, 1992). In particular, the tyrphostin class of PTK inhibitors have shown great promise in *in vitro* antiproliferative and receptor phosphorylation studies (Levitzki and Gazit, 1995; Levitzki, 1990). The reversible nature of receptor tyrosine kinase inhibition may necessitate frequent drug administration schedules to achieve a continuous inhibition of the growth-factor stimulated proliferation. Therefore, *in vivo* characterisation of tyrphostin disposition is required to design schedules that would deliver sustained plasma and tissue levels. This report is the first to characterise the *in vivo* disposition of a tyrphostin PTK inhibitor.

RG13022 was rapidly absorbed after injection and was detectable at the earliest measured time point (2 min). The

**Figure 2** The concentration – time profile of RG13022 after a single intraperitoneal injection of 20 mg kg$^{-1}$ in MF1 *nu*/*nu* mice. Each data point represents the pooled sample from three mice.

rapid absorption could be due to the hydrophobic nature and low molecular weight of the compound and may be influenced by the vehicle (DMSO). The peak plasma level was 6.34 $\mu g\ ml^{-1}$ or 28 $\mu M$. This concentration is 2.5-fold higher than the $IC_{50}$ of RG13022-associated inhibition of HN5 DNA synthesis after a 24 h incubation. The concentrations needed for *in vivo* activity of tyrphostins are not known. It has been reported that $IC_{50}$ values for the antiproliferative activity of tyrphostins in whole organ culture are 5–10 times higher than in cell culture (Dvir *et al.*, 1991). Nevertheless, our results, together with the report of RG13022 *in vivo* activity, indicate that pharmacologically relevant concentrations are achievable *in vivo* (Yoneda *et al.*, 1991). The terminal half-life of RG13022, determined from plasma samples measured over 90 min, was estimated to be 50.4 min. Although optimum assessment of terminal elimination is made after drug measurement over a 4–5 half-life time interval, RG13022 was not detectable in plasma by 2 h post injection. Therefore, it is unlikely that sufficient quantity of drug was present for continuous inhibition of receptor tyrosine kinase activity. However, caution should be applied with the extrapolation of plasma tyrphostin exposure as a surrogate marker of tumour exposure. The pharmacokinetic profile of tumour RG13022 is not known as tissue levels were not evaluated.

Together with RG13022, three other drug-related substances were observed, with the largest peak eluting at 9 min. No standards were available, and therefore these substances could not be quantified. One of these drug-related substances showed the chromatographic and spectral characteristics of the pp. Extensive NMR spectroscopy and chromatographic analysis identified the pp as the (*E*) isomer of RG13022. The product was rapidly formed *in vivo* and had a terminal $t_{1/2}$ of 23.9 min. It is not possible to deduce from these data if the formation of this substance *in vivo* occurred before the absorption of the parent drug from the peritoneum or within the plasma. The pp was not detectable in the drug solution used for *in vivo* studies. The plasma profiles of the two other drug-related peaks reflected the production of metabolites. An initial increase in plasma concentration of these metabolites was followed by a biexponential decline. Formation of these substances before absorption, followed by a slower absorption profile than RG13022, is also possible. However, substances with similar chromatographic and spectral characteristics were not seen *in vitro* experiments (unpublished observations).

Yoneda *et al.* (1991) mentioned that RG13022 undergoes light-induced isomerisation, but assumed that the two isomeric forms were equally active. This contrasts with the

**Figure 3** Inhibition of (**a**) [$^3$H]thymidine incorporation and (**b**) cytotoxicity of RG13022 (○) and its (*E*) isomer (●) in HN5 cells after 24 h incubation.

**Figure 4** The influence of RG13022 on *in vivo* growth of HN5 xenografts following 400 $\mu g$ daily for 21 days after (**a**) the implantation of tumour or (**b**) tumours had reached a volume of 50 $mm^3$. ■, RG13022; □, vehicle.

results of the current work, in which the (*E*) isomer was found to be one third as potent as the (*Z*) isomer (RG13022) inhibiting HN5 DNA synthesis after 24 h *in vitro* incubation. Therefore, evaluation of RG13022 disposition alone will give an underestimate of the agent's systemic exposure.

In contrast to the findings of Yoneda *et al.* (1991), RG13022 demonstrated no anti-tumour activity against human tumour xenografts in the current study. The absence of tumour growth suppression was found when RG13022 was administered chronically for 21 days from the day of tumour inoculation or after tumour establishment had occurred. Although both studies used squamous cell carcinoma cell lines with high levels of EGFR, differences in cell signalling, downstream targets or death pathways between the cell lines may contribute greatly to the *in vivo* activity of RG13022 previously described (Modjtahedi *et al.*, 1993; Yoneda *et al.*, 1991), However, previous studies have demonstrated *in vivo* inhibition of HN5 tumour growth by EGFR antibodies (Modjtahedi *et al.*, 1993). This demonstrates that HN5 cells are susceptible to growth inhibition by targeting the EGFR. An additional difference between the study of Yoneda *et al.* (1991) and the current study was the drug administration schedule. Yoneda *et al.* 1991 administered RG13022 at 200 $\mu$g per mouse twice daily (400 $\mu$g total dose), whereas in the current study we administered the same dose as a single daily injection. The rapid elimination of RG13022 resulted in plasma concentrations $<1$ $\mu$M by 20 min after injection. The rate of drug degradation of other tyrphostin compounds has also been shown to influence the degree of *in vitro* PTK inhibitory activity against $pp60^{c\text{-}src}$ and EGFR (Ramdas *et al.*, 1994). The antiproliferative activity of RG13022 has an $IC_{50}$ of 2–20 $\mu$M against human cancer cell lines after prolonged (>16 h) *in vitro* incubations (data not shown) (Reddy *et al*,, 1992; Yoneda *et al.*, 1991). In addition, although RG13022 had potent inhibitory activity against HN5 fell DNA synthesis, little cytotoxic activity was observed for either RG13022 or its geometric isomer. This suggests that a better administration schedule for RG13022 would include frequent injections or continuous administration. In the current study twice daily administration of RG13022 was not tolerated by the MF1 *nu*/*nu* mice because of the toxicity of the DMSO drug vehicle (data not shown). An improved formulation for RG13022 is therefore required before the *in vivo* activity of this PTK inhibitor can be assessed further. The results also highlight the need to develop more water-soluble tyrosine kinase inhibitors that give sustained plasma and tissue exposures, as continuous blockage of the tyrosine kinase signal is likely to be required for growth inhibition.

**Acknowledgements**
This work was supported by the Cancer Research Campaign (UK). Dr McLeod was the recipient of a NCI-EORTC Exchange Award. Dr Lear was the recipient of a Glasgow University (Senate) Scholarship. Professor Workman acknowledges the award of a Cancer Research Campaign Life Fellowship.

### References

AARONSON SA. (1991). Growth factors and cancer. *Science*, **154**, 1146–1153.

BARTLETT JMS, LANGDON SP, SIMPSON BJB, STEWART M, KATSAROS D, SISMONDI P, LOVE S, SCOTT WN, WILLIAMS ARW, LESSELLS AM, MACLEOD KG, SMYTH JF AND MILLER WR. (1996). The prognostic value of epidermal growth factor receptor mRNA expression in primary ovarian cancer. *Br. J. Cancer*, **73**, 301–306.

BRUNTON VG AND WORKMAN P. (1993). Cell signalling targets for antitumor drug development. *Cancer Chemother. Pharmacol.*, **32**, 1–19.

BRUNTON VG, LEAR MJ, ROBINS DJ, WILLIAMSON S AND WORKMAN P. (1994). Synthesis and antiproliferative activity of tyrphostins containing heteroaromatic moieties. *Anti-cancer Drug Design*, **9**, 291–309.

CHAKRABARTY S, RAJAGOPAL S AND HUANG S. (1995). Expression of antisense epidermal growth-factor receptor RNA down modulates the malignant behavior of human colon cancer cells. *Clin. Exp. Metastasis*, **13**, 191–195.

DVIR A, MILNER Y, CHOMSKY O, GILON C, GASIT A AND LEVITZKI A. (1991). The inhibition of EGF-dependent proliferation of keratinocytes by tyrphostin tyrosine kinase blockers. *J. Cell. Biol.*, **113**, 857–865.

FAN Z, BASELGA J, MASUI H AND MENDELSOHN J. (1993). Antitumor effect of anti-epidermal growth factor receptor monoclonal antibodies plus cis-diamminedichloroplatinum on well established A431 cell xenografts. *Cancer Res.*, **53**, 4637–4642.

FRY DW, KRAKER AJ, MCMICHAEL A, AMBROSO LA, NELSON JM, LEOPOLD WR, CONNORS RW AND BRIDGES AJ. (1994). A specific inhibitor of the epidermal growth factor receptor tyrosine kinase. *Science*, **265**, 1093–1095.

HARRIS AL. (1994). What is the biological, prognostic, and therapeutic role of the EGF receptor in human breast cancer. *Breast Cancer Res. Treat.*, **29**, 1–2.

HARRIS AL. (1990). The epidermal growth factor receptor as a target for therapy. *Cancer Cells*, **2**, 321–323.

LEAR MJ, MUIR KW, ROBINS DJ, RYCROFT DS AND TORABI AA. Synthesis, interconversion, and X-ray crystal structures of (*E*)- and (*Z*)-2-(3′-Pyridyl)-3-(3,4-dimethoxyphenyl)propenonitrile. *J. Chem. Soc. Perkin. Trans. II*, submitted.

LEVITZKI A. (1990). Tyrphostins – potential antiproliferative agents and novel molecular tools. *Biochem. Pharmacol.*, **40**, 913–918.

LEVITZKI A AND GAZIT A. (1995). Tyrosine kinse inhibition: an approach to drug development. *Science*, **267**, 1782–1788.

MENDELSOHN J. (1990). The epidermal growth factor receptor as a target for therapy with antireceptor monoclonal antibodies. *Semin. Cancer Biol.*, **1**, 339–344.

MODJTAHEDI H, ECCLES S, BOX G, STYLES J AND DEAN C. (1993). Immunotherapy of human tumour xenografts overexpressing the EGF receptor with rat antibodies that block growth factor–receptor interaction. *Br. J. Cancer*, **67**, 254–261.

MORONI MC, WILLINGHAM MC AND BEGUINOT L. (1992). EGF-R antisense RNA blocks expression of the epidermal growth-factor receptor and suppresses the transforming phenotype of a human carcinoma cell line. *J. Biol. Chem.*, **267**, 2714–2722.

PLUMB JA, MILROY R AND KAYE SB. (1989). Effects of the pH dependence of 3-(4,5-dimethylthiazol-2-yl)-2,5-diphenyltetrazolium bromide-formazan absorption on chemosensitivity determined by a novel tetrazolium-based assay. *Cancer Res.*, **49**, 4435–4440.

RAMDAS L, MCMURRAY JS AND BUDDE RJA. (1994). The degree of inhibition of protein tyrosine kinase activity by tyrphostin 23 and 25 is related to their instability. *Cancer Res.*, **54**, 867–869.

REDDY KB, MANGOLD GL, TANDON AK, YONEDA T, MUNDY GR, ZILBERSTEIN A AND OSBORNE CK. (1992). Inhibition of breast cancer cell growth in vitro by a tyrosine kinase inhibitor. *Cancer Res.*, **52**, 3636–3641.

STERN DF, HARE DL, CECCHINI MA AND WEINBERG RA. (1987). Construction of a novel oncogene based on synthetic sequences encoding epidermal growth factor. *Science*, **235**, 321–324.

TRAXLER P, TRINKS U, BUCHDUNGER E, METT H, MEYER T, MULLER M, REGENASS U, ROSEL J AND LYDON N. (1995). [(Alkylamino)methyl]acrylophenones: potent and selective inhibitors of the epidermal growth factor receptor protein tyrosine kinase. *J. Med. Chem.*, **38**, 2441–2448.

ULLRICH A AND SCHLESSINGER J. (1990). Signal transduction by receptors with tyrosine kinase activity. *Cell*, **61**, 203–212.

WARD WHJ, COOK PN, SLATER AM, DAVIES DH, HOLDGATE GA AND GREEN LR. (1994). Epidermal growth factor receptor tyrosine kinase. Investigation of catalytic mechanism, structure-based searching and discovery of a potent inhibitor. *Biochem. Pharmacol.*, **48**, 659–666.

WORKMAN P, BRUNTON VG AND ROBINS DJ. (1992). Tyrosine kinase inhibitors. *Semin. Cancer Biol.*, **3**, 369–381.

WRIGHT C, ANGUS B, NICHOLSON S, SAINSBURY JRC, CAIRNS J, GULLICK WJ, KELLY P, HARRIS AL AND HORNE CHW. (1989). Expression of c-*erb*B-2 oncoprotein: a prognostic indicator in human breast cancer. *Cancer Res.*, **49**, 2087–2090.

YONEDA T, LYALL RM, ALSINA MM, PERSONS PE, SPADA AP, LEVITZKI A, ZILBERSTEIN A AND MUNDY GR. (1991). The antiproliferative effects of tyrosine kinase inhibitors tyrphostins on a human squamous cell carcinoma in vitro and in nude mice. *Cancer Res.*, **51**, 4430–4435.